# Contributing reviewers 2023

**DOI:** 10.1186/s40942-023-00514-4

**Published:** 2023-12-30

The Editorial team is grateful to the dedicated reviewers who helped them improve the quality of the articles published from January 2023 until November 2023 within the journal International Journal of Retina and Vitreous.

Luis Abad

Maaly Abdel Fattah

Ahmed Abdelshafy

Antoaneta Adzic Zecevic

Kunihiko Akiyama

Ahmed Roshdy Alagorie

Giuseppe Maria Albanese

Valeria Albano

Hasenin Al-Khersan

David Almeida

Gabriel Andrade

Yusuke Arai

Cengiz Aras

Sruthi Arepalli

Gerd Auffarth

Neil Avery

Alay Banker

Aditya Bansal

Gabriel Barbosa

Victor Bellanda Candido Ferreira

Vinicius Bergamo

Frederico Bermudes

Chhaya Bharti

Laurentino Biccas Neto

Alper Bilgic

Lukas Bisorca-Gassendorf

Marcio Bittar Nehemy

Rocio Blanco-Garavito

Maria Teresa Bonanomi

David Boyer

Rodrigo Brant Fernandes

Oswaldo Brasil

Thiago Cabral

Leandro Cabral Zacharias

Pedro Carricondo

Shuo Chen

Yu-Bai Chou

Felipe Conti

Giulia Corradetti

Leonardo Cunha

Francyne Cyrino

Francesco Maria D’Alterio

Eduardo Damasceno

Francisco Max Damico

Sabin Dang

Hudson De Carvalho Nakamura

Sandro de Zanet

Kushal Delhiwala

Lucas Denadai

Elona Dhrami-Gavazi

Rodrigo Donato Costa

Susan Dounce

Lilianne Duarte

Pedro Duraes Serracarbassa

Ayman Elnahry

Geoffrey Emerson

Rania Estawro

Teodoro Evans

Wael Ewais

Lorenzo Fabozzi

Joao Felix

Arthur Fernandes

André Freitas

Paula Fukuhara

Minoru Furuta

Gerardo Garcia-Aguirre

Thomas Gardner

Marco Gioia

Roger Goldberg

Andrzej Grzybowski

Kaan Gündüz

Yuki Hashimoto

John Helal Junior

Kenzo Hokazono

Josef Huemer

Mark Humayun

Yasuko Ikegami

Yasuko Ikegami

Tatsuya Inoue

Makoto Inoue

David Isaac

Kai Jin

Eliane Jorge

Wajiha Kheir

Benjamin Kim

Vinicius Kniggendorf

Vinod Kumar

Pradeep Kumar

Lik Thai Lim

Luiz Lima

Mateus Lins

Anat Loewenstein

Jose Lucas de Souza Filho

Luiz Lucatto

Mathew MacCumber

Cleide Machado

Fernando Malerbi

Ahmad Mansour

Virgínia Mares

João Pedro Marques

Akiyama Masato

Thais Mendes

Carsten Meyer

Zofia Michalewska

Andrew Mihalache

Nilva Simeren Bueno De Moraes Ambrogini

Luis Nakayama

Raja Narayanan

Heloisa Nascimento

Mauricio Nascimento

Carlos Neto

Massimo Nicolò

Mohamad Reza Niyuosha

Joshua Ong

Renato Passos

Felipe Pereira

Fernanda Porto

Rony Preti

David Ramsey

Zsuzsa Recsan

Caio Regatieri

Francisco Rodriguez

Alejandro Rodriguez-Garcia

Jaime Roizenblatt

Marina Roizenblatt

Mariana Rossi Thorell

Kumar Saurabh

Sandeep Saxena

Anastasios Sepetis

Dhaivat Shah

Ashish Sharma

Tarun Sharma

Helio Shiroma

Paolo Silva

Bradley Smith

Scott Sonne

Osias Souza

Eduardo Tomazoni

Andrew Tsai

Mudit Tyagi

Maria Vadalà

Raul Velez-Montoya

Carlos Veloso

Pradeep Venkatesh

Victor Villegas

Lihteh Wu

Tae Keun Yoo

Young Yoon

